# Supplementary figures and images for: Recommendation of Tahiti acid lime cultivars through Bayesian probability models
Source: PLoS One. 2024 Mar 5;19(3):e0299290. doi: 10.1371/journal.pone.0299290 (PMC10914267; doi:10.1371/journal.pone.0299290)

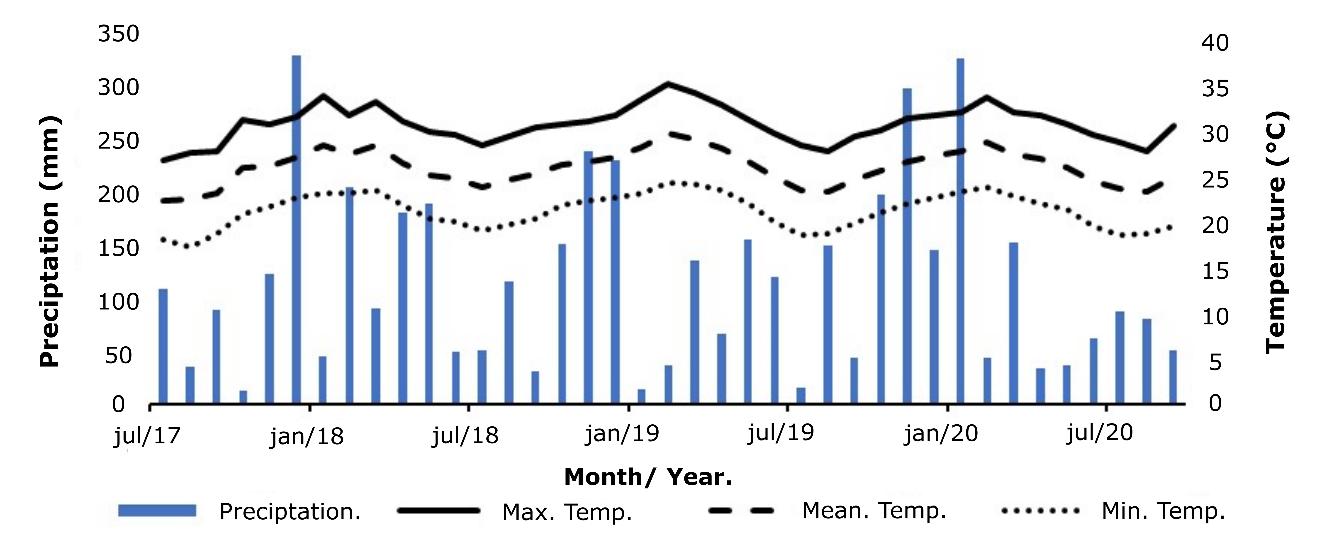

Supplement: S1 Fig — (TIF) [file pone.0299290.s001.tif]

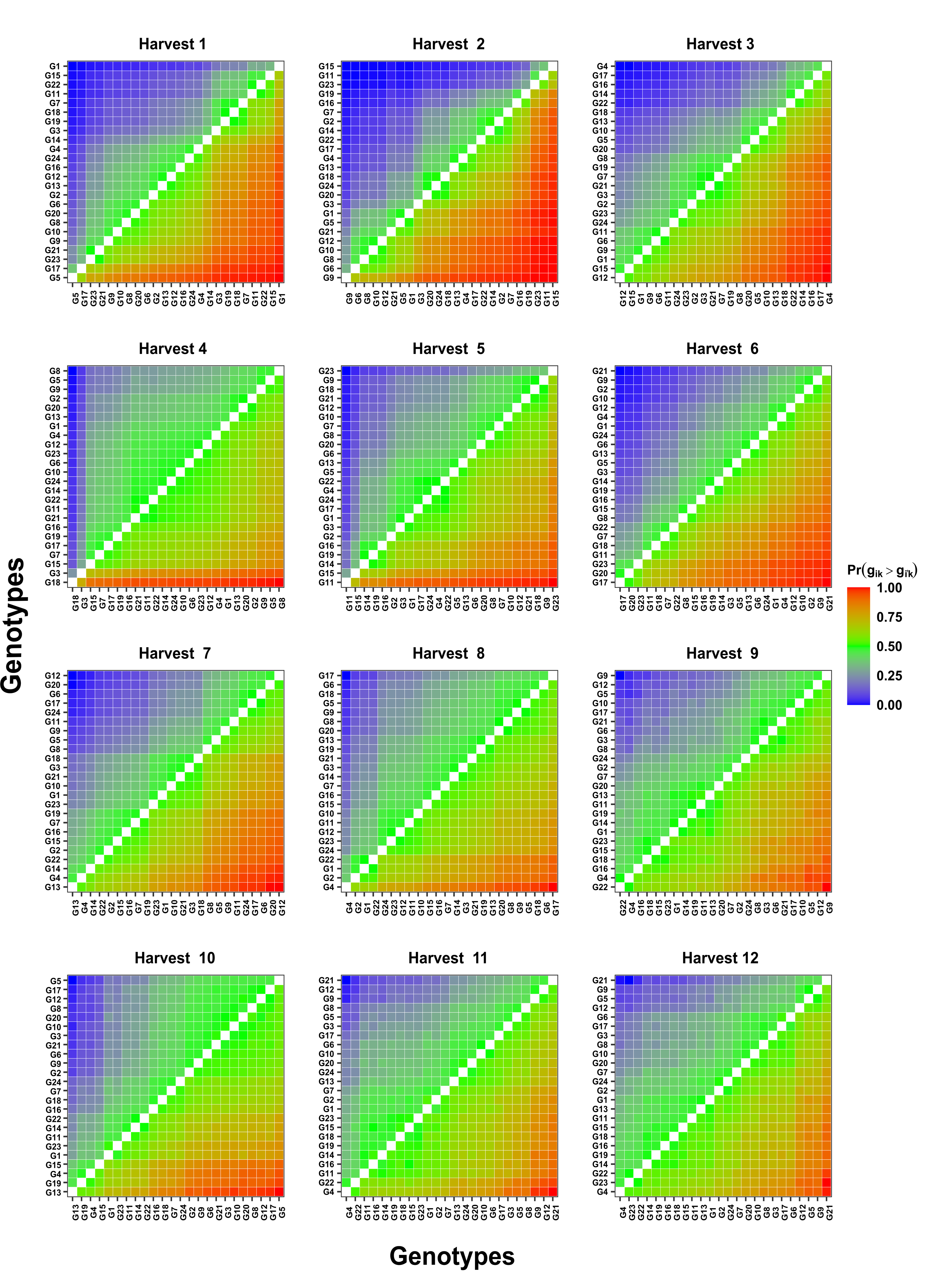

Supplement: S2 Fig — (TIF) [file pone.0299290.s002.tif]
