# Supplementary material for: Recommendation of Tahiti acid lime cultivars through Bayesian probability models
Source: PLoS One. 2024 Mar 5;19(3):e0299290. doi: 10.1371/journal.pone.0299290 (PMC10914267; doi:10.1371/journal.pone.0299290)
Supplement: Attachment — Submitted filename: PONE-D-23-38484_reviewed.pdf [file pone.0299290.s004.pdf]

PLOS ONE

Application of Bayesian probabilistic models for recommendation of ‘Tahiti’ acid lime using longitudinal data

--Manuscript Draft--

Manuscript Number:	PONE-D-23-38484
Article Type:	Research Article
Full Title:	Application of Bayesian probabilistic models for recommendation of ‘Tahiti’ acid lime using longitudinal data
Short Title:	Application of Bayesian models in 'Tahiti' acid lime
Corresponding Author:	Leonardo Lopes Bhering<br>Universidade Federal de Vicosa<br>Viçosa, MG BRAZIL
Keywords:	Citrus latifolia, fruit tree breeding, perennial crops, Hamiltonian Monte Carlo, repeated measures, genotype-by-harvest interaction
Abstract:	Probabilistic models that predict genotype superiority and stability have potential in a breeding program of perennial species. These models identify superior and persistent individuals using probability theory, providing a measure of uncertainty that can aid the recommendation. The objective of our study was to evaluate the use of Bayesian probabilistic models for the recommendation of superior and persistent genotypes of ‘Tahiti’ acid lime evaluated in 12 harvests. Leveraging the Monte Carlo Hamiltonian sampling algorithm, we calculated the probability of superior performance (superior genotypic value), and the probability of superior stability (reduced variance of the genotype-by-harvests interaction) of each genotype. The probability of superior stability was compared to a measure of persistence estimated from genotypic values predicted using a frequentist model. Our results demonstrated the applicability and advantages of the Bayesian probabilistic model, yielding similar parameters to those of the frequentist model, while providing further information about the probabilities associated with genotype performance and stability. Genotypes G15, G4, G18, and G11 emerged as the most superior in performance, whereas G24, G7, G13, and G3 were identified as the most stable. This study highlights the potential of Bayesian probabilistic models in assisting the selection in perennial species breeding programs
Order of Authors:	Renan Garcia Malikouski
	Filipe Manoel Ferreira
	Saulo Fabrício da Silva Chaves
	Evellyn Gisele de Oliveira Couto
	Kaio Olimpio das Graças Dias
	Leonardo Lopes Bhering
Additional Information:	
**Question**	**Response**
**Financial Disclosure**<br><br>Enter a financial disclosure statement that describes the sources of funding for the work included in this submission. Review the submission guidelines for detailed requirements. View published research articles from *PLOS ONE* for specific examples.	The author(s) received no specific funding for this work.

Powered by Editorial Manager® and ProduXion Manager® from Aries Systems Corporation

This statement is required for submission and **will appear in the published article** if the submission is accepted. Please make sure it is accurate.<br><br>**Funded studies**<br>Enter a statement with the following details:<br>• Initials of the authors who received each award<br>• Grant numbers awarded to each author<br>• The full name of each funder<br>• URL of each funder website<br>• Did the sponsors or funders play any role in the study design, data collection and analysis, decision to publish, or preparation of the manuscript?<br><br>Did you receive funding for this work?	
**Competing Interests**<br><br>Use the instructions below to enter a competing interest statement for this submission. On behalf of all authors, disclose any competing interests that could be perceived to bias this work—acknowledging all financial support and any other relevant financial or non-financial competing interests.<br><br>This statement is required for submission and **will appear in the published article** if the submission is accepted. Please make sure it is accurate and that any funding sources listed in your Funding Information later in the submission form are also declared in your Financial Disclosure statement.<br><br>View published research articles from *PLOS ONE* for specific examples.	The authors have declared that no competing interests exist.

Powered by Editorial Manager® and ProduXion Manager® from Aries Systems Corporation

**NO authors have competing interests**<br><br>Enter: *The authors have declared that no competing interests exist.*<br><br>**Authors with competing interests**<br><br>Enter competing interest details beginning with this statement:<br><br>*I have read the journal's policy and the authors of this manuscript have the following competing interests: [insert competing interests here]*<br><br>* typeset	
**Ethics Statement**<br><br>Enter an ethics statement for this submission. This statement is required if the study involved:<br><br>• Human participants<br>• Human specimens or tissue<br>• Vertebrate animals or cephalopods<br>• Vertebrate embryos or tissues<br>• Field research<br><br>Write "N/A" if the submission does not require an ethics statement.<br><br>General guidance is provided below. Consult the submission guidelines for detailed instructions. **Make sure that all information entered here is included in the Methods section of the manuscript.**	N/A

Powered by Editorial Manager® and ProduXion Manager® from Aries Systems Corporation

**Format for specific study types**<br><br>**Human Subject Research (involving human participants and/or tissue)**<br>• Give the name of the institutional review board or ethics committee that approved the study<br>• Include the approval number and/or a statement indicating approval of this research<br>• Indicate the form of consent obtained (written/oral) or the reason that consent was not obtained (e.g. the data were analyzed anonymously)<br><br>**Animal Research (involving vertebrate animals, embryos or tissues)**<br>• Provide the name of the Institutional Animal Care and Use Committee (IACUC) or other relevant ethics board that reviewed the study protocol, and indicate whether they approved this research or granted a formal waiver of ethical approval<br>• Include an approval number if one was obtained<br>• If the study involved *non-human primates*, add *additional details* about animal welfare and steps taken to ameliorate suffering<br>• If anesthesia, euthanasia, or any kind of animal sacrifice is part of the study, include briefly which substances and/or methods were applied<br><br>**Field Research**<br><br>Include the following details if this study involves the collection of plant, animal, or other materials from a natural setting:<br>• Field permit number<br>• Name of the institution or relevant body that granted permission	
**Data Availability**<br><br>Authors are required to make all data underlying the findings described fully available, without restriction, and from the time of publication. PLOS allows rare exceptions to address legal and ethical concerns. See the PLOS Data Policy and FAQ for detailed information.	No - some restrictions will apply

A Data Availability Statement describing where the data can be found is required at submission. Your answers to this question constitute the Data Availability Statement and **will be published in the article**, if accepted.<br><br>**Important:** Stating 'data available on request from the author' is not sufficient. If your data are only available upon request, select 'No' for the first question and explain your exceptional situation in the text box.<br><br>Do the authors confirm that all data underlying the findings described in their manuscript are fully available without restriction?	
**Describe where the data may be found in full sentences. If you are copying our sample text, replace any instances of XXX with the appropriate details.**<br><br>• If the data are **held or will be held in a public repository**, include URLs, accession numbers or DOIs. If this information will only be available after acceptance, indicate this by ticking the box below. For example: *All XXX files are available from the XXX database (accession number(s) XXX, XXX.*).<br>• If the data are all contained **within the manuscript and/or Supporting Information files**, enter the following: *All relevant data are within the manuscript and its Supporting Information files.*<br>• If neither of these applies but you are able to provide **details of access elsewhere**, with or without limitations, please do so. For example:<br><br>*Data cannot be shared publicly because of [XXX]. Data are available from the XXX Institutional Data Access / Ethics Committee (contact via XXX) for researchers who meet the criteria for access to confidential data.*<br><br>*The data underlying the results presented in the study are available from (include the name of the third party*	Data cannot be shared publicly because of the studies were conducted on a private agricultural property. Data are available from the Renan Malikouski Institutional Data Access / Ethics Committee (contact via renan_malikouski@hotmail.com) for researchers who meet the criteria for access to confidential data.<br><br>The data underlying the results presented in the study are available from (include the name of the third party and contact information or URL).

and contact information or URL).<br>• This text is appropriate if the data are owned by a third party and authors do not have permission to share the data.<br><br>* typeset	
Additional data availability information:	Tick here if the URLs/accession numbers/DOIs will be available only after acceptance of the manuscript for publication so that we can ensure their inclusion before publication.

Powered by Editorial Manager® and ProduXion Manager® from Aries Systems Corporation

Manuscript
Click here to access/download;Manuscript;Manuscript_Prob_Lima_Final.doc

MANUSCRIPT

Application of Bayesian probabilistic models for recommendation of 'Tahiti' acid lime using longitudinal data

Application of Bayesian models in 'Tahiti' acid lime

Renan Garcia Malikouski[1], Filipe Manoel Ferreira[2], Saulo Fabrício da Silva Chaves[3], Evellyn Giselly de Oliveira Couto[3], Kaio Olimpio das Graças Dias[1], Leonardo Lopes Bhering[1]*

[1] Departamento de Biologia Geral, Universidade Federal de Viçosa, Viçosa, Minas Gerais, Brazil.

[2] Department of Crop Science - College of Agricultural Sciences, São Paulo State University, Botucatu, São Paulo, Brazil.

[3] Departamento de Agronomia, Universidade Federal de Viçosa, Viçosa, Minas Gerais, Brazil.

*Corresponding author. E-mail: leonardo.bhering@ufv.br (LLB)

All authors contributed equally to this work.

Abstract

Probabilistic models that predict genotype superiority and stability have potential in a breeding program of perennial species. These models identify superior and persistent individuals using probability theory, providing a measure of uncertainty that can aid the recommendation. The objective of our study was to evaluate the use of Bayesian probabilistic models for the recommendation of superior and persistent genotypes of ‘Tahiti’ acid lime evaluated in 12 harvests. Leveraging the Monte Carlo Hamiltonian sampling algorithm, we calculated the probability of superior performance (superior genotypic value), and the probability of superior stability (reduced variance of the genotype-by-harvests interaction) of each genotype. The probability of superior stability was compared to a measure of persistence estimated from genotypic values predicted using a frequentist model. Our results demonstrated the applicability and advantages of the Bayesian probabilistic model, yielding similar parameters to those of the frequentist model, while providing further information about the probabilities associated with genotype performance and stability. Genotypes G15, G4, G18, and G11 emerged as the most superior in performance, whereas G24, G7, G13, and G3 were identified as the most stable. This study highlights the potential of Bayesian probabilistic models in assisting the selection in perennial species breeding programs.

Keywords: *Citrus latifolia*, fruit tree breeding, perennial crops, Hamiltonian Monte Carlo, repeated measures, genotype-by-harvest interaction

Introduction

Breeding perennial fruit crops presents a unique set of challenges. The long juvenile phase and the variable expression of quantitative traits over time can delay and

mislead the selection of superior genotypes [1]. This differential performance over time can be a reflex of the genotypes-by-harvests interaction (G[illegible]. Therefore, in perennial fruit breeding, repeated measures on the same plant over time are mandatory, which increases costs and the duration of breeding cycl[illegible]]. In the presence of GHI, breeders must expand their goals beyond performance evaluation to also consider the stability of the genotypes over harvests [3–5].

In 'Tahiti' acid lime (*Citrus latifolia* Tanaka L.), several strategies have been proposed to address the challenges related to the extended juvenile phase and the presence of GHI. Grafting, a well-established technique in citrus propagation, influences vegetative growth and shortens the juvenile phase, facilitating earlier evaluations in breeding programs [6,7]. Furthermore, research has demonstrated that employing repeatability models allows for accurate genotype selection in 'Tahiti' acid lime after just four measurements [8]. To address the common issue of non-orthogonal longitudinal data, resulting from the interdependence of measurements taken on the same individual over time, approaches like random regression models using Legendre orthogonal polynomials have been applied [9]. Random regression models enable the estimation of the genotypic trajectory of evaluated treatments over time. Despite considerable progress in 'Tahiti' acid lime breeding, straightforward methodologies for exploring GHI and identifying high-performance and stable genotypes are yet to be established[illegible]

A recent methodology proposed by Dias et al. [10] can help to untangle genotype-by-environment interaction by using probability theory. This method aims to reduce the risk associated with the selection of a given genotype, which is the daily dilemma of the farmers, who seek to guide their actions to minimize the risks of low production for a given crop [1[illegible]. Dias et al. [10] proposed to use probability concepts from Bayesian models to assist in the selection of genotypes that gather favorable alleles for performance

and stability across environments. Furthermore, it allows a straightforward recommendation based on the probability of a given genotype to be selected considering its performance and stability, and a pairwise comparison of the probabilities of the evaluated selection candidates. This methodology has been proposed for the multiple-location context. Nevertheless, we believe that the same ideas are valid for the multi-harvest scenario. In this paper, we evaluate the use of Bayesian probabilistic models for the recommendation of superior and persistent genotypes of ‘Tahiti’ acid lime.

Materials and Methods

Trial and plant material

We evaluated 24 combinations of rootstock and scion of ‘Tahiti’ acid lime for fruit yield, expressed in kg of fruit per plant (Kg/tree). The hybrids *Citrumelo swingle* (*Citrus paradisi* X *Poncirus trifoliata*) and *Citrandarin ‘riverside’* (*Citrus sunki* X *Poncirus trifoliata*) were used as rootstock for scions of 12 clones of ‘Tahiti’ acid lime (Table 1). The plant materials came from the Active Germplasm Bank of Embrapa Mandioca e Fruticultura [12]. Each combination was considered a different selection candidate. The trial was laid out in a complete randomized block design, with four replications. Each plot was composed of three plants. The inter-rows and inter-columns spacing was 6 x 3 m, respectively. We performed the analysis described in the next topic using the plots mean.

The trial was established in July 2015, in São Mateus municipality, Espírito Santo, Brazil (18°48'21"S, 39°53'30"W, 35 m of altitude). This region has a rainy season in summer and a dry season in winter, being classified as Aw, following the classification of Köppen [13]. The precipitation and temperature during the period of the experiment is illustrated in the Fig S1 of the Supplementary Material. The data was collected between

July 2017 and September 2020, and consisted of 12 harvests carried out in the following days after planting: 736, 808, 861, 918, 972, 1083, 1200, 1249, 1415, 1568, 1633 and 1867.

Table 1. Code for 24 combinations of rootstock and scion of 'Tahiti' acid lime.

Scion	Rootstock	
	Citrumelo swingle	*Citrandarin riverside*
Bello Fruit	G1	G13
Eledio	G2	G14
Iconha	G3	G15
Itarana	G4	G16
Santa Rosa	G5	G17
Bearss Lime	G6	G18
CNPMF01	G7	G19
CNPMF02	G8	G20
CNPMF2001	G9	G21
CNPMF5059	G10	G22
BRS Passos	G11	G23
Persian 58	G12	G24

Statistical analyses

We applied the probabilistic approach of Dias et al. [10] by fitting two Bayesian models using the the *rstan* package [14] and ProbBreed package [15]. The first model had a homogeneous residual variance (B-ID) and presented the following conditional normal probability:

$$y_{ijk} N(E[y_{ijk}], \sigma)$$

where:

$$E[y_{ijk}] = \mu + g_i + r_j + h_k + gh_{ik} + e$$

in which $E[y_{ijk}]$ is the expectation of the phenotype from the ith genotype, evaluated in the jth block, at the kth harvest; μ is the overall mean; g_i is the genotypic effect; r_j is the block effect; h_k is the harvest effect; and gh_{ik} is the genotypes-by-harvests interaction.

The prior probability distribution of each parameter of the model was defined as:

$$\mu \sim N(0, \sigma_{[\mu]})$$

$$r \sim N(0, \sigma_{[r]})$$

$$h \sim N(0, \sigma_{[h]})$$

$$g \sim N(0, \sigma_{[g]})$$

$$gh \sim N(0, \sigma_{[gh]})$$

$$e \sim HalfCauchy(0, \sigma_{[e]})$$

$$\sigma \sim HalfCauchy(0, \sigma_{[\alpha]})$$

where $N(0, \sigma_{[\alpha]})$ and $HalfCauchy(0, \sigma_{[\alpha]})$ represent the normal and half-Cauchy distributions, respectively, with mean equal to zero and with different $\sigma^2_{[\alpha]}$ scale parameters. The following hyperpriors were considered for the respective parameters:

$$\sigma_{[\mu]} \sim HalfCauchy(0, \varphi)$$

$$\sigma_{[r]} \sim HalfCauchy(0, \varphi)$$

$$\sigma_{[h]} \sim HalfCauchy(0, \varphi)$$

$$\sigma_{[g]} \sim HalfCauchy(0, \varphi)$$

$$\sigma_{[gh]} \sim HalfCauchy(0, \varphi)$$

where φ represents a predetermined global hyperparameter($\varphi = max(y) * 10$), defined in such a way that results in a weakly informative second level hyperpriors. Therefore, the data dominated the posterior distributions [16]. The half-Cauchy distribution is restricted to positive values, being often recommended as a prior distribution when modeling variance parameters [17].

The second Bayesian model had heterogeneous residual variances (B-DG). This model has the same considerations of the M-ID, except that $\sigma_k Half Cauchy(0, \sigma_{[\sigma_k]})$. We selected the best-fitted model via the Watanabe-Akaike Information Criterion 2 (WAIC2) [16]. We used a Monte Carlo Hamiltonian algorithm in four Markov chains with 4000 samples and 50% burn-in.

Convergence diagnostics

The scale reduction factor ($\hat{R}$) was used to assess the effectiveness of the convergence of the Markov chain Monte Carlo (MCMC). This metric indicates whether the chains have mixed sufficiently, and the estimated model parameters have reached a stable distribution. The closer the $\hat{R}$ to 1, the greater the quality of mixing and convergence [16]. Greater values imply that more iterations are needed.

We also conducted a graphical analysis to visually assess how well the data generated by our model aligns with the true generative process of the observed data. This involved creating samples (referred to as "y_{gen}") from the fitted models using ancestral sampling from the conditional joint distribution and then plotting these samples against the observed data. Additionally, we employed posterior predictive p-values to gauge how closely the statistical measures (maximum, minimum, median, mean, and standard deviation) of the data generated by the fitted models resembled those of the observed data [16]. For instance, when considering the maximum statistic, we defined the Bayesian p-value as follows: $p_{max} = Pr(T(y_{gen}, \theta) \geq T(y, \theta) \mid y)$, where T is the statistic test. The degree of similarity between the statistics derived from the generated data and those from the observed data increases as the Bayesian p-values approach 0.5.

Probability of superior performance and genotypic stability

The probability metrics for both performance and stability utilized in this study were proposed by Dias et al. [10] and implemented in the ProbBreed R software [15]. Aiming to select the top four genotypes, we sampled the posterior distribution of the marginal genotypic values given the observed phenotypic values. In each sample, we ranked these genotypes in descending order of posterior genotypic values (g_i^s). Then, we counted the number of events where a given genotype appeared in the subset of superior genotypes (Ω), i.e., among the top four (selection intensity of 16%). The probability of superior performance of a genotype is given by the number of samples it appeared in divided by the total number of samples. In summary:

$$Pr(g_i^s \epsilon \Omega_s | y) = \frac{1}{S} \sum_{s=1}^{S} T(g_i^s \epsilon \Omega_s | y)$$

where S $(s = 1,2, \dots, S)$ is the number of samples and $I(g_i^s \in |y)$ is an indicator variable that maps failure (0, if $g_i^s \notin$) or success (1, if $g_i^s \in$).

We calculated the stability across harvests based on the variance of the effect of GHI. Genotypes with lower GHI variance $(var[gh_{ik}])$ tend to be more stable, showing less fluctuation in their performance between harvests. One can draw a parallel of this metric and the frequentist persistence (see the next topic). Following the same idea described in the last paragraph, we built an indicator variable to map the success of a given candidate belonging to the subset of the top four genotypes with smaller $var[gh_{ik}]$ $(I(var[gh_{ik}]^s \in \ell V | y))$. The probability of superior stability is given as follows:

$$Pr(var(gh_i) \in \ell V | y) = \frac{1}{S} \sum_{s=1}^{S} I(var[gh_i]^s \in \ell V | y)$$

We used the ideas previously described for the two probabilities to perform pairwise comparisons between selection candidates. The goal is to investigate the chances

of a given genotype being superior, whether in performance or stability, to its peers. The pairwise probabilities of superior performance and the pairwise probabilities of superior stability are given by, respectively:

$$Pr(g_i > g_{i'}|y) = \frac{1}{S}\sum_{s=1}^{S} I\,(g_i^s > g_{i'}^s|\,y)$$

and

$$Pr(var(gh_i) < var(gh_{i'})|y) = \frac{1}{S}\sum_{s=1}^{S} I(var[gh_i]^s < var[gh_{i'}]^s\,|y)$$

where $I(g_i^s > g_{i'}^s|\,y)$ is an indicator variable mapping success if g_i^s has higher genotypic value than $g_{i'}^s$, or failure otherwise; and $I(var[gh_i]^s < var[gh_{i'}]^s\,|y)$ is another indicator variable mapping success if gh_{ik} has lower variance than $gh_{i'k}$, or failure otherwise.

The probability of superior performance within harvests and the pairwise probability of superior performance within harvests can be obtained by the following equations, respectively:

$$Pr(g_{ik} > g_{i'k}|y) = \tfrac{1}{S}\textstyle\sum_{s=1}^{S} I\left(g_{ik}^s > g_{i'k}^s|\,y\right),$$

$$Pr(var(gh_{ik}) < var(gh_{i'k})\,|y) = \frac{1}{S}\sum_{s=1}^{S} I(var[gh_{ik}]^s < var[gh_{i'k}]^s\,|y)$$

where $I\left(g_{ik}^s > g_{i'k}^s|\,y\right)$, is an indicator variable mapping success if g_i^s has higher genotypic value than $g_{i'}^s$ in the harvest k, or failure otherwise; and $I(var[gh_{ik}]^s < var[gh_{i'k}]^s\,|y)$ is another indicator variable mapping success if gh_{ik} has lower variance than $gh_{i'k}$ in harvest k, or failure otherwise.

Stability in the frequentist context

To compute the stability, we first fitted the following frequentist model (F-DG):

$$y = 1\mu + X_1 r + X_2 h + Z_1 g + Z_2 gh + Z_3 p + e$$

where y is the vector of phenotypic data, 1 is a vector of ones, μ is the intercept of the model, r is the vector of repetition effects (assumed to be fixed), h is the vector of harvest effects (assumed to be fixed), g is the vector of genotypic effects (assumed to be random) $[g \sim N(0, I\sigma_g^2)]$, gh is the vector of the effects of the genotype-by-harvest interaction (assumed to be random) $[gh \sim N(0, I\sigma_{gh}^2)]$, p is the vector of permanent environmental effect (assumed to be random) $[p \sim N(0, I\sigma_p^2)]$, and e is the vector of residues associated with phenotypic observations (random) $[e \sim N(0, R)]$, where $\boldsymbol{R}$ is a residual covariance matrix. The capital letters X_1 and X_2 refer to the incidence matrix for the fixed effects, and Z_1, Z_2 and Z_3 are the incidence matrix for the random effects of the respective effects.

We estimated the variance components and predicted the genetic values using the residual maximum likelihood – REML [18], and the best linear unbiased predictor – BLUP [19] , respectively. The significance tests of the random effects were verified via likelihood ratio test (LRT) [20].

A concept analogous to stability called "persistence" is used by breeders of perennial forages, and refers to the ability to survive and keep producing dry matter for long periods [21,22]. In perennial fruit plants, this concept can be readjusted as the ability to maintain a high fruit yield for several years [23]. Therefore, persistence is analogous to an ecological stability of the genotypes (P_i) based on the distance between each genotype in relation to the ideotype. The ideotype (g_{max}) was defined as the maximum genotypic value estimated on each harvest. We used the following expression to estimate the persistence [22]:

$$P_i = \frac{\frac{1}{\sum_{k=1}^{12}(g_i - g_{\max})^2}}{\sum_{i=1}^{24}\frac{1}{\sum_{k=1}^{12}(g_i - g_{max})^2}}$$

We compared the genotype ranking of the Bayesian probabilistic model and persistence via the frequentist model using Spearman correlation

We performed the analysis using *R* software environment, version 4.2.1 [24]. The Bayesian models were fitted using the probabilistic programming language *Stan* [25] from the *rstan* package [14] , and the ProbBreed R package [15]. We fitted the linear mixed models using *ASReml-R* (version 4.1) [26].

Results

Probability of superior performance of genotypes

The Bayesian models (B-ID and B-DG) displayed a mean value of the statistic $\hat{R}$ close to 1, indicating strong convergence of the model parameters (Table 2). Notably, among these models, the B-DG model exhibited the best fit, as evidenced by its lower WAIC2 value (Table 2). Note how the density of the data generated by B-DG model follows the same trend as the density of the real data. This indicates the model's effectiveness in replicating the distribution of observed data through the generated data, highlighting its reliability in capturing the underlying patterns (Fig 1A). Considering the findings from the B-DG, the posterior distribution of genotypic values of the 24 genotypes exhibited a variable overlapping pattern among their highest posterior density intervals (Fig 1B).

Table 2. Comparative statistics of Bayesian models using the 'Tahiti' acid file dataset.

Predictive a posteriori verification statistic	Homogeneous residual variance model	Heterogeneous (diagonal) residual variance model
WAIC2	8420.87	6899.61
$\hat{R}$	1.00	0.99

Where WAIC2 is the Watanabe–Akaike information criterion, and $\hat{R}$ is the potential scale reduction factor.

Fig 1. Bayesian distribution of the observed and generated data of the 'Tahiti' acid lime dataset **(A)**. Caterpillar plot of the genotypic posterior effects (and their 95% and 97.5% HPDs, represented by the thick and thin lines, respectively) of 24 genotypes of posterior effects **(B)**. Marginal probability of superior performance of the 24 genotypes **(C)**. Probability of superior stability of the 24 genotypes (**D)**.

Genotypes G15, G4, G18 and G11 presented the highest posterior genotypic values (Fig 1B) and the highest probability of superior performance (Fig 1C). Genotypes G15, G4, G18, G11, G3, G23, G22, G1, G19, G14, G2, G13 and G16 were selected at least in a few samples, while the remaining 11 genotypes did not appear among the selected in any sample (Fig1C). Genotypes G15 and G4 offers low risk of bad performance if selected (probability of superior performance equal to or higher than 75%). Genotypes G24 and G7 had the highest probability of superior stability, meaning that they have the less variable performance across harvests (Fig 1D).

The pairwise comparison graph presents two symmetrical sides, which indicate the probability of success (lower diagonal) and failure (upper diagonal) of the genotypes in the *x*-axis being superior to the ones of the *y*-axis (Fig 2A). G15, for example, has a high probability of beating all genotypes. On the other hand, G9 is beaten by all its peers, except for G21, which wins three-thirds of the time. The greenish color indicates

genotypes that tie in their performance (probability close to 50%), like G18 and G11, G2 and G14, and G10 and G24 (Fig 2A).

Fig 2. Pairwise probability of superior performance among genotypes (A). Probabilities of superior performance within environments (B).

The probabilities of a given genotype belonging to the group of selected ones in each harvest varies for high performance genotypes (Fig 2B). Genotypes G15, G4, G18, and G11 consistently displayed probabilities exceeding 50% across nearly all harvests. Conversely, genotypes G9 and G21 exhibited nil probabilities throughout all harvests, implying that they are not recommended due to their consistently poor performance (Fig 2B). According to the probability of superior performance within the harvests, high production by certain genotype in one harvest did not guarantee the same level of performance in subsequent harvests (Supplementary material - Fig S2).

Probability of genotypic stability

The probability of encountering a 'Tahiti' acidic lime genotype with minimal variation for stability was generally low. Only G24 exhibited values exceeding 0.3, indicating it to be the most stable genotype among those under evaluation (Fig 1D). The frequentist model (F-DG) demonstrated the significance of GHI according to the LRT. Also, the variance components exhibited similar magnitudes for both models (Table 3). The σ_g^2overshadowed the σ_{gh}^2 and σ_p^2, with values of 2.20 for the F-DG model and 2.44 for the B-DG. The residual variance displayed varying values across different harvests, ranging from 0.88 to 412.17 in the F-DG and from 0,63 to 384.17 in the B-DG (Table 3).

Table 3. Variance components estimates for a frequentist heterogeneous (diagonal) residual variance model (F-DG) and a Bayesian heterogeneous (diagonal) residual variance model (B-DG)

to a 'Tahiti' acid lime dataset. For the Bayesian model, it includes the corresponding lower (L) and upper (U) highest posterior density (HPD), considering a confidence level α = 0.05.

Parameter		F-DG	B-DG		
		Component	L-HPD	Component	U-HPD
σ_g^2 σ_{gh}^2		2.21	1.31	2.44	4.22
σ_p^2		0.81	0.44	0.76	1.09
σ_e^2	H1	1.19	0.89	1.24	1.67
	H2	0.88	0.63	0.91	1.29
	H3	0.86	0.70	0.97	1.33
	H4	5.60	4.48	5.88	7.69
	H5	3.07	2.72	3.60	4.72
	H6	4.22	3.37	4.52	5.99
	H7	10.26	8.53	11.23	14.78
	H8	20.17	20.57	26.71	34.32
	H9	412.17	328.66	418.67	531.69
	H10	136.74	113.61	146.63	186.56
	H11	404.45	324.73	413.08	521.54
	H12	473.61	384.17	492.71	621.24

σ_g^2: genotypic variance, σ_{gh}^2 is the genotypes-by-harvesters interaction, σ_p^2 is the variance of the environmental permanent effect, and σ_e^2 is the residual variance for the 12 harvesters (H1 to H12).

Persistence in the frequentist sense presented different results from the probability of superior stability of Bayesian models. However, in both contexts, the values were low. Genotypic persistence in the frequentist context ranged from 5.7 to 3.4 (Fig 3). Except for G3, which presented the highest value and well above the others, the other genotype values were in a range of , showing the low ability of the F-DG to distinguish the persistence of these genotypes. The correlation between the persistence rankings in the Bayesian context (Fig 1D) and those in the frequentist context (Fig 3) exhibited a

coefficient of 0.69. This suggests a statistically significant correlation between the classifications provided by the two methods, considering a confidence level of $\alpha = 0.05$.

Fig 3. Persistence of 24 genotypes of 'Tahiti' acid lime via the frequentist model with diagonal residual variance.

Discussion

The consideration of the GHI is very informative in the assessment of perennial species. This is because gene expression varies in response to environmental factors across different harvests [27]. The model selection based on WAIC2, coupled with the observed increase in residual variance over successive harvests, provides robust evidence in favor of the suitability of the heterogeneous model for data fitting over the homoscedastic model. These findings underscore the critical importance of appropriate modeling and accounting for diverse sources of variation in longitudinal datasets.

Comparing the genotypic values associated with a posteriori probability ensures greater confidence in the analysis of the performance of the ‘Tahiti’ acid lime genotypes. Bayesian probability measures offer breeders the opportunity to delve into the probability of a particular genotype surpassing others, including scenarios where a candidate genotype may outperform a widely adopted cultivar [10]. This probability-based approach aids decision-making, especially when the difference between genotypes’ performance is small. These probabilities are dependent on the selection intensity, a value that is often predefined in breeding programs, depending on the stage [28]. Thence, probabilities are an intuitive metric and offer an enhanced reliability for recommendations, since it provides information about the risks. Its application simplifies

decision-making processes and opens avenues for application in various domains beyond plant breeding [10].

Genotypes with the highest probability of superior performance, namely G15, G4, G18, and G11, emerged as strong candidates for recommendation. These genotypes, as determined through probabilistic methods, possess alleles that impart adaptation to the changes in environmental conditions encountered through the harvests, maintaining consistently good performance. Indeed, the probability of superior performance is a measure of stability in an agronomic sense. The presence of GHI imposes changes for the selection of superior genotypes based on a single or a few harvests. Therefore, considering the genotype`s performance across multiple harvests is advisable for making well-informed decisions when selecting genetically superior candidates.

Initially employed in forage species to assess the maintenance of productivity levels through multiple cuts [22], the concept of persistence is also relevant in the context of perennial fruit crops. We can make a parallel between persistence and the probability of superior stability, as both represent ecological stability, i.e., invariance of performance. In the Bayesian framework, the probability of being selected among the four most stable genotypes was, in general, low (values below 0.4). This metric had a 50% agreement rate in identifying the top four most persistent genotypes with the frequentist persistence. Both approaches selected G24 and G3 among the four most persistent genotypes.

Certain advantages of the Bayesian model deserve attention. The incorporation of priors enhances confidence in selecting materials with varying levels of persistence [10,29]. Furthermore, Bayesian models offer the advantage of obtaining variance components with associated high probability density intervals. These credibility intervals provide a more intuitive means of quantifying component uncertainty. Also, from an asymptotic perspective, Bayesian credibility intervals outperform frequentist confidence

intervals [16], since frequentist confidence intervals may prove inaccurate for small or moderate sample sizes and may, in certain instances, fail to converge to the true parameter value as the sample size increases [30]. Likewise in mixed model, Bayesian models work well in common situation of plant breeding, such as unbalanced data, heterogeneous residual variance [5,31,32].

Conclusion

By applying probabilistic Bayesian models in longitudinal `Tahiti` acid lime dataset, we estimated the probability of superior performance of a genotype and the pairwise probabilities of superior performance between genotypes for both across and within harvests. Genotypes G15, G4, G18 and G11 were considered superior, and genotypes G24, G7, G13 and G3 were considered the most stable ones. Therefore, we believe that Bayesian probabilistic models can assist to more accurate recommendation in perennial fruit crops evaluated along many harvests, since it allows a more direct and precise interpretation of the performance and persistence of the candidate's genotypes.

Acknowledgments

The authors are grateful to the Coordenação de Aperfeiçoamento de Pessoal de Nível Superior (Capes, Code 001), Conselho Nacional de Desenvolvimento Científico e Tecnológico (CNPq), Fundação de Amparo à Pesquisa do Estado de Minas Gerais (Fapemig) and Fundação de Amparo à Pesquisa do Estado do Espírito Santo (Fapes). Filipe Manoel Ferreira was supported by FAPESP (São Paulo Research Foundation, Grant 2023/04881-3),

References

1. Gray SB, Brady SM. Plant developmental responses to climate change. Dev Biol. 2016;419: 64–77. doi:10.1016/j.ydbio.2016.07.023

2. Chaves SFS, Dias LAS, Alves RS, Alves RM, Evangelista JSPC, Dias KOG. Leveraging multi-harvest data for increasing genetic gains per unit of time for fruit yield and resistance to witches' broom in Theobroma grandiflorum. Euphytica. 2022;218: 171. doi:10.1007/s10681-022-03126-5

3. Zhang S, Liang M, Wang N, Xu Q, Deng X, Chai L. Reproduction in woody perennial Citrus: an update on nucellar embryony and self-incompatibility. Plant Reprod. 2018;31: 43–57. doi:10.1007/s00497-018-0327-4

4. Rai MK, Shekhawat NS. Recent advances in genetic engineering for improvement of fruit crops. Plant Cell, Tissue and Organ Culture (PCTOC). 2014;116: 1–15.

5. Malosetti M, Ribaut J-M, van Eeuwijk FA. The statistical analysis of multi-environment data: modeling genotype-by-environment interaction and its genetic basis. Front Physiol. 2013;4. doi:10.3389/fphys.2013.00044

6. García-Muñoz MC, Henao-Rojas JC, Moreno-Rodríguez JM, Botina-Azain BL, Romero-Barrera Y. Effect of rootstock and environmental factors on fruit quality of Persian lime (Citrus latifolia Tanaka) grown in tropical regions. Journal of Food Composition and Analysis. 2021;103: 104081.

7. Hassanzadeh Khankahdani H, Rastegar S, Golein B, Golmohammadi M, Aboutalebi Jahromi A. Effect of rootstock on vegetative growth and mineral elements in scion of different Persian lime (Citrus latifolia Tanaka) genotypes. Sci Hortic. 2019;246: 136–145. doi:10.1016/j.scienta.2018.10.066

8. Malikouski RG, Peixoto MA, de Morais AL, Elizeu AM, Zucoloto M, Bhering LL. Repeatability coefficient estimates and optimum number of harvests in graft/rootstock combinations for'tahiti'acid lime. Acta Sci Agron. 2021;43: e51740–e51740.

9. Malikouski RG, Alves RS, Peixoto MA, Ferreira FM, do Nascimento EF, de Morais AL, et al. Selection index based on random regression model in 'Tahiti' acid lime. Euphytica. 2022;218: 153. doi:10.1007/s10681-022-03105-w

10. Dias KOG, dos Santos JPR, Krause MD, Piepho H-P, Guimarães LJM, Pastina MM, et al. Leveraging probability concepts for cultivar recommendation in multi-environment trials. Theoretical and Applied Genetics. 2022;135: 1385–1399. doi:10.1007/s00122-022-04041-y

11. Eskridge KM, Byrne PF, Crossa J. Selection of stable varieties by minimizing the probability of disaster. Field Crops Res. 1991;27: 169–181.

12. Santos MG, Passos OS, Soares Filho WS, Girardi EA, Gesteira AS, Ferreira CF. Variability analysis of "Persian" acid lime tree selections using agronomic and

molecular markers. Genetics and Molecular Research. 2013;12: 4604–4614. doi:10.4238/2013.October.17.3

13. Alvares CA, Stape JL, Sentelhas PC, Gonçalves JL de M. Modeling monthly mean air temperature for Brazil. Theor Appl Climatol. 2013;113: 407–427. doi:10.1007/s00704-012-0796-6

14. Stan Development T. RStan: the R interface to Stan. R package version 217 3. 2018.

15. Chaves SF da S, Dias. Kaio Olimpio das Graças, Krause MD. ProbBreed: Probability Theory for Selecting Candidates in Plant Breeding. 2023.

16. Gelman A, Carlin JB, Stern HS, Rubin DB. Bayesian Data Analysis, Chapman & Hall/CRC Texts in Statistical Science Vol. 3. 2013.

17. Gelman A. Prior distributions for variance parameters in hierarchical models (comment on article by Browne and Draper). Bayesian Anal. 2006;1. doi:10.1214/06-BA117A

18. Patterson HD, Thompson R. Recovery of inter-block information when block sizes are unequal. Biometrika. 1971;58: 545–554. doi:10.1093/biomet/58.3.545

19. Henderson CR. Best Linear Unbiased Estimation and Prediction under a Selection Model. Biometrics. 1975;31: 423. doi:10.2307/2529430

20. Wilks SS. The Large-Sample Distribution of the Likelihood Ratio for Testing Composite Hypotheses. The Annals of Mathematical Statistics. 1938;9: 60–62. doi:10.1214/aoms/1177732360

21. Parsons AJ, Edwards GR, Newton PCD, Chapman DF, Caradus JR, Rasmussen S, et al. Past lessons and future prospects: plant breeding for yield and persistence in cool-temperate pastures. Grass and Forage Science. 2011;66: 153–172. doi:10.1111/j.1365-2494.2011.00785.x

22. Rocha JRASC, Machado JC, Carneiro PCS. Multitrait index based on factor analysis and ideotype-design: proposal and application on elephant grass breeding for bioenergy. GCB Bioenergy. 2018;10: 52–60. doi:10.1111/gcbb.12443

23. Chaves SFS, Evangelista JSPC, Alves RS, Ferreira FM, Dias LAS, Alves RM, et al. Application of linear mixed models for multiple harvest/site trial analyses in perennial plant breeding. Tree Genet Genomes. 2022;18: 44.

24. R Development Core Team. R: A language and environment for statistical computing. Vienna, Austria: R Foundation for Statistical Computing; 2020.

25. Carpenter B, Gelman A, Hoffman MD, Lee D, Goodrich B, Betancourt M, et al. Stan: A Probabilistic Programming Language. J Stat Softw. 2017;76. doi:10.18637/jss.v076.i01

26. Butler DG, Cullis BR, Gilmour AR, Gogel BJ, Thompson R. ASReml-R reference manual version 4. VSN International Ltd, Hemel Hempstead, HP1 1ES, UK. 2017.

27. Ferreira FM, de Carvalho Rocha JR do AS, Alves RS, Elizeu AM, Benites FRG, de Resende MDV, et al. Estimates of repeatability coefficients and optimum number of measures for genetic selection of Cynodon spp. Euphytica. 2020;216: 70. doi:10.1007/s10681-020-02605-x

28. Silva ÉDB da, Xavier A, Faria MV. Impact of Genomic Prediction Model, Selection Intensity, and Breeding Strategy on the Long-Term Genetic Gain and Genetic Erosion in Soybean Breeding. Front Genet. 2021;12. doi:10.3389/fgene.2021.637133

29. Silva JO da C, Bruckner CH, Carneiro PCS, de Resende MDV, Alves RS, Matias RGP. Estimates of genetic parameters and repeatability coefficients in S2 peach genotypes grown in a subtropical environment. 2020.

30. Kruschke J. Doing Bayesian data analysis: A tutorial with R, JAGS, and Stan. 2014.

31. Smith AB, Cullis BR, Thompson R. The analysis of crop cultivar breeding and evaluation trials: an overview of current mixed model approaches. J Agric Sci. 2005;143: 449–462.

32. Crossa J. From genotype x environment interaction to gene x environment interaction. Curr Genomics. 2012;13: 225–244.

Supporting Information

Fig S1. Climatic data of precipitation (mm) and temperature (ºC) from July 2017 to July 2020 in the field trial location.

Fig S2. Pairwise probability of superior performance among genotypes within harvest.

Fig1

Click here to access/download;Figure;Figure1.png

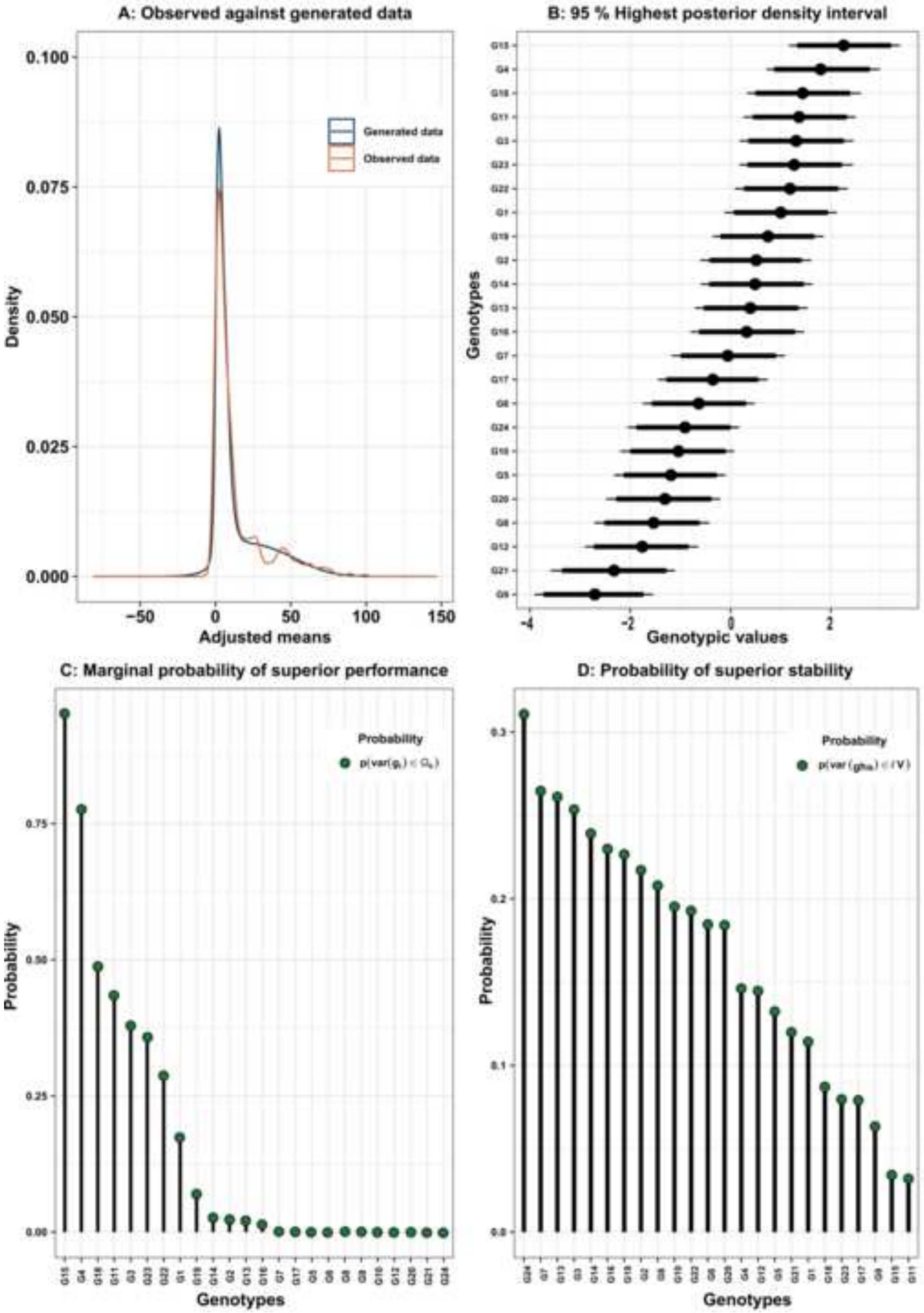

Fig2

Click here to access/download;Figure;Fig2.tiff

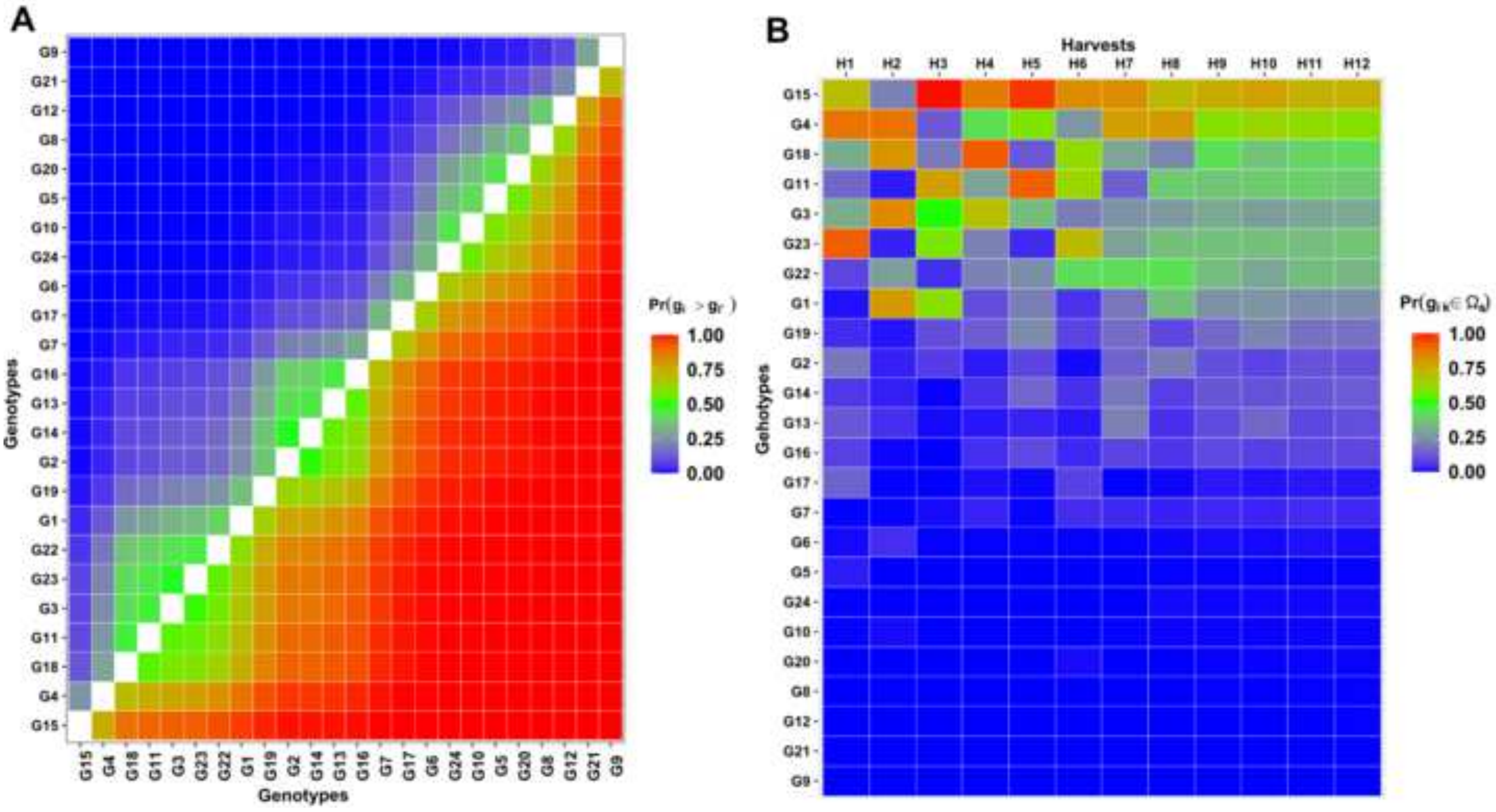

Fig3

Click here to access/download;Figure;Fig3.tiff

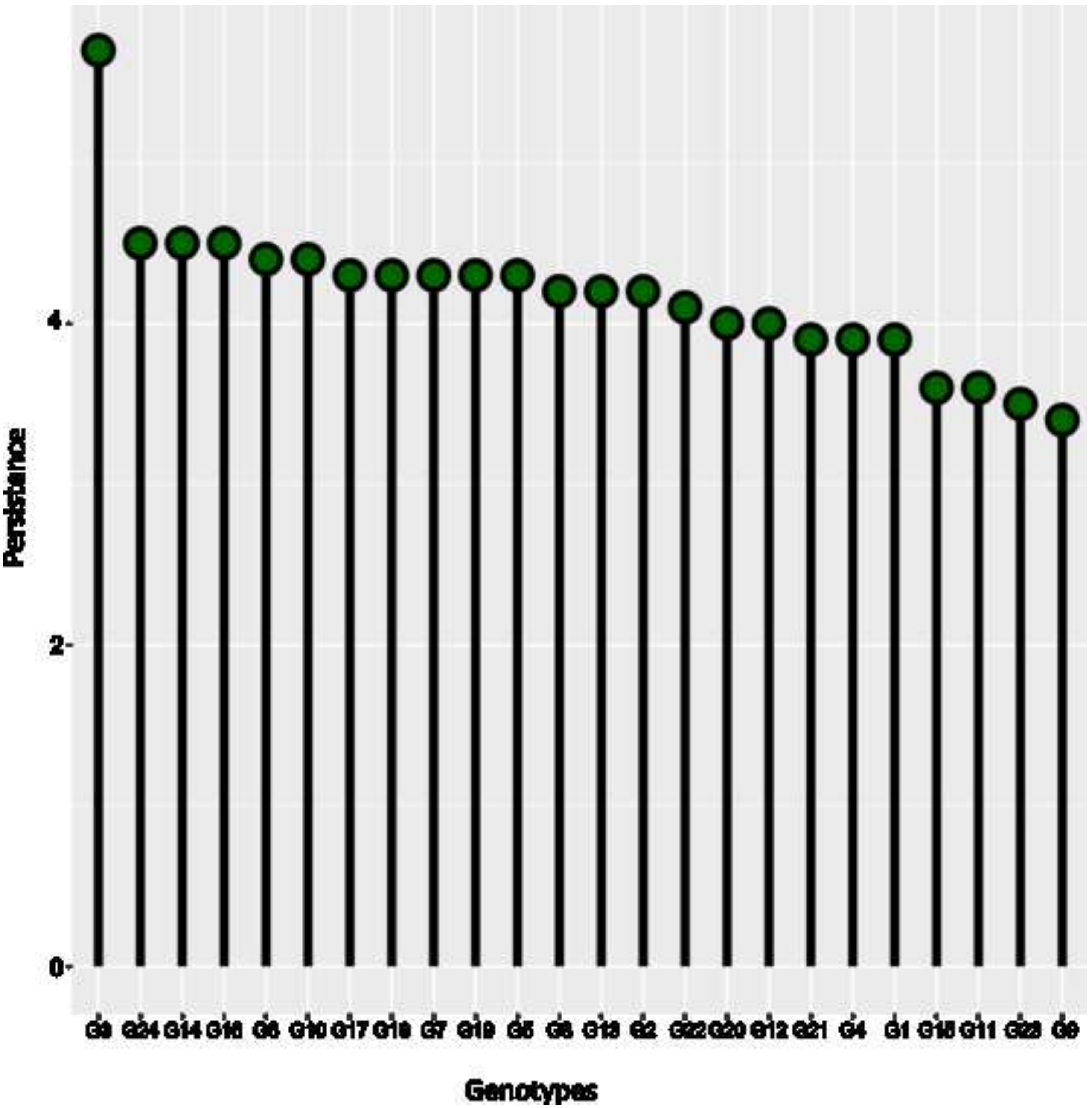

Click here to access/download
Supporting Information - Compressed/ZIP File Archive
Supporting Information.zip
